# The use of virtual tools in narrowing the impact of health disparities in neurology

**DOI:** 10.3389/fped.2022.1028833

**Published:** 2022-10-14

**Authors:** Jean-Baptiste Le Pichon, Stephanie Horton, Omar Abdelmoity, Mark A. Hoffman, Emily Cramer, Nirmeen Kishk, Salah Hamada, Ahmed Abdelmoity

**Affiliations:** ^1^ Division of Neurology, Department of Pediatrics, Children’s Mercy Hospital, Kansas City, MO, United States; ^2^ Washington University at St. Louis, Saint Louis, MO, United States; ^3^ Department of Pediatrics, Children’s Mercy Research Institute, Kansas City, MO, United States; ^4^ Division of Health Services / Outcomes Research, Children’s Mercy Research Institute, Kansas City, MO, United States; ^5^ Department of Neurology, Cairo University, Giza, Egypt; ^6^ Department of Neurosurgery, Ain Shams University, Cairo, Egypt

**Keywords:** neurology, education, epilpesy, virtual medicine, telehealth, treatment gap

## Abstract

The concept of Epilepsy Treatment Gap (ETG) refers to the proportion of people with epilepsy who are not being appropriately treated. The ETG in the USA approaches 10%, with historically underserved populations and rural populations disproportionately affected. The ETG in Low-and Middle-Income Countries (LMIC) is reported to be 5–10 times higher than in high-income countries. The growing availability of reliable internet access offers a unique opportunity to provide better care to children and adults with epilepsy. In this paper we explore various telehealth (TH) initiatives that have leveraged the availability of easy and free access to an internet connection in reducing the ETG in underserved regions of the world. We describe several interventions targeted to reach patients and providers in rural areas of the United States and in LMIC. First, we examine initiatives that were developed to improve patient access to coordinated care and education regarding epilepsy and seizures. Next, we describe an intervention designed to improve knowledge of epilepsy diagnosis and treatment for providers in LMIC. We conclude with a brief overview of the use of virtual tools in diminishing the ETG.

## Introduction

Despite improvements in care for epilepsy, the global burden of epilepsy remains high with approximately 46 million people affected in 2016 (1). In 2001 the International League Against Epilepsy introduced the concept of Epilepsy Treatment Gap (ETG) as the proportion of people with epilepsy who are not being appropriately treated (2). The ETG in the USA approaches 10%. However, there is evidence that the ETG is higher in historically underserved populations (3) and in rural populations (4). Furthermore, while improvements in care have reduced the burden of epilepsy in developed countries, the burden remains high in Low-And-Middle-Income-Countries (LMIC)- where multiple barriers prevent patients from receiving effective treatments, including counterproductive cultural beliefs and practices, and lack of access to physicians with a basic knowledge of epilepsy diagnosis and treatment (1). It is estimated that about three quarters of people with epilepsy living in LMIC don't get necessary treatment (5). Epilepsy is most prevalent in children younger than five and among older adults (6). The burden of disease is especially heavy in children and adults with breakthrough seizures. However, it is estimated that up to 70% of patients with epilepsy can become seizure free with proper antiepileptic treatment (5). The growing availability of reliable internet access offers a unique opportunity to utilize telehealth (TH) services to provide better care to children and adults with epilepsy. In this paper we explore various initiatives that have leveraged the availability of easy and free access to an internet connection in reducing the ETG in underserved regions of the world. We will describe several interventions that were targeted to reach patients and providers, in rural areas of the United States, and in LMIC. In the first part of the paper, we will examine initiatives that were developed to improve patient access and knowledge, while in the second half we will describe an intervention designed to improve knowledge of epilepsy diagnosis and treatment for providers.

## Reaching the patient

With the COVID19 pandemic, the use of TH in the United States became a necessity. Out of this necessity, general acceptance of telemedicine has grown, including in the management of pediatric epilepsy (7). With the increase of TH services, new applications have arisen to improve access and to leverage the power of synchronous and asynchronous virtual connections to improve education for patients, families, and caregivers in LMIC (8). One way in which this technology has naturally evolved is in the care of complex patients living in remote areas. In this first example we show how remote connections *via* TH can be used to manage even the most complex patients.

### Leveraging virtual communications to provide expert advice

The first example is a case report that demonstrates how one can leverage the power of TH to care for complex patients in remote areas. In July 2021 one of our neurology providers (JBLP) was approached by a provider working in the Kurdistan region of Iraq, asking for assistance with the care of a 9-month-old infant with a complex medical history. The child was the mother's sixth pregnancy. Four of the children (two girls and two boys) died early in life (between 45 days of life and 3 years old); at least one of these children had a history of epilepsy. The other sibling is a healthy 9-year-old boy. The patient, an infant girl, was born full term without complications. She was hospitalized at 1 month of age with cold like symptoms. A head CT revealed “brain atrophic changes”. She developed failure-to-thrive and seizure like activity. Several EEGs showed both focal and generalized epileptiform activity. The team in Iraq was able to obtain exome testing in Germany that revealed a homozygous likely pathogenic variant in the *C2orf69* gene, consistent with a genetic diagnosis of the autosomal-recessive *C2orf69*-related neurodevelopmental-disorder. Variants in *C2orf69* associate with mitochondrial respiratory enzyme chain dysfunction. This rare disorder has been reported in less than a dozen patients, most of middle eastern ancestry (Turkish, Pakistani, and Kurdish) (9). At the time of the consultation, the child was treated with levetiracetam and phenobarbital, she struggled with feeding, had developed failure to thrive, and was having daily breakthrough seizures. With the help of a dietician from the Charlie Foundation and a Neurologist with experience in the management of mitochondrial diseases, both in the United States, the team in Iraq was able to initiate a ketogenic diet. Several meetings were arranged using synchronous Zoom videoconferencing for the team in the United States to meet the child and the family. The child achieved ketosis and since starting the diet has been stable with rare breakthrough seizures and good weight gain. Clinically she is more alert and interactive. The team continues to follow the child with regular updates and lab results using the WhatsApp messaging application. This example illustrates how by leveraging the power of virtual communications, providers in LMIC can reach out for consultations for patients with rare and complex metabolic disorders (in this case a mitochondrial disease) and successfully manage these children.

### YouTube as an educational instrument

In the second example we demonstrate the asynchronous use of YouTube to facilitate patient and family education and engagement in the care of children and youth with epilepsy (CYE). Family engagement has been shown to have significant impacts on the care of children with chronic diseases, including improved compliance with medication regimen, disease outcome, and quality of life (10). Family engagement is contingent on a basic understanding of the illness that affects the children.

#### Method

We created a series of 17 videos in English and 17 videos with identical content in Spanish (Table 1). The videos are approximately 2–3 min in length and are publicly available on YouTube. We also targeted patients with epilepsy and/or seizures seen in the Neurology Clinic at Children's Mercy Kansas City. To leverage these videos, a quality improvement project was initiated in the department to “prescribe” videos to patients, based on their specific disease and duration. This was printed in the patient depart paperwork with a QR code that took them to a REDCap survey including: demographics, a pre-test, the prescribed video(s), and a post-test. This study was completed as part of the REACT project (Reaching out for Epilepsy in Adolescents and Children through Telemedicine), sponsored by a Health Resources and Services Administration (HRSA) grant. The YouTube Channel hosting the REACT videos was established on August 7, 2020, the first videos were initially made public on March 11, 2021. Advertisements are suppressed. A web page describing the REACT program, including video descriptions and the YouTube links to the videos, was added to the Children's Mercy web site (https://tinyurl.com/25dwyc9n). We monitored overall traffic to the videos to assess response utilizing YouTube analytics for the period from March 11, 2021 to May 22, 2022. We focused on the channel overview and the video with the highest viewership. For that video, we reviewed audience retention patterns (dashboard view), traffic source and device type.

**Table 1:** Epilepsy videos and number of families who completed each video.

Video #	Educational video	Number completed
	**GROUP 1**	
1	What is epilepsy?	10
2	What is a seizure?	12
3	What's the difference between a seizure and epilepsy?	6
4	Are there different kinds of seizures?	6
5	Are there things that can increase the chances that my child will have a seizure?	5
6	What should I do if my child has a seizure?	2
7	Are there precautions I need to take for my teen?	3
16	Do kids with epilepsy have other symptoms?	3
17	FAQs about epilepsy	3
	**TOTAL COMPLETION GROUP 1**	50
	**GROUP 2**	
8	How do you diagnose epilepsy?	0
9	How do medications treat epilepsy?	1
10	What is neurostimulation and how does it treat epilepsy?	3
11	What is ROSA and how does it treat epilepsy?	1
12	Can you treat epilepsy with surgery?	1
13	What is the ketogenic diet, and does it treat epilepsy?	1
14	**TOTAL COMPLETION GROUP 2**	7
	**GROUP 3**	
14	What is a tonic-clonic seizure?	1
15	What is an absence seizure?	5
	**TOTAL COMPLETION GROUP 3**	6

This table represents the 17 videos that were created by the Children's Mercy Hospital Team. The number of completions indicates the number of families who completed the demographics, pre survey, video and post survey. It is further broken down into 3 groups. Group 1 includes 9 videos that provide general information regarding seizures and epilepsy. Group 2 includes 6 videos which discuss diagnoses, treatment plans, and nonpharmacological therapies. Finally, Group 3 includes 2 videos which are specific to seizure types.

#### Results

**Figure 1:**
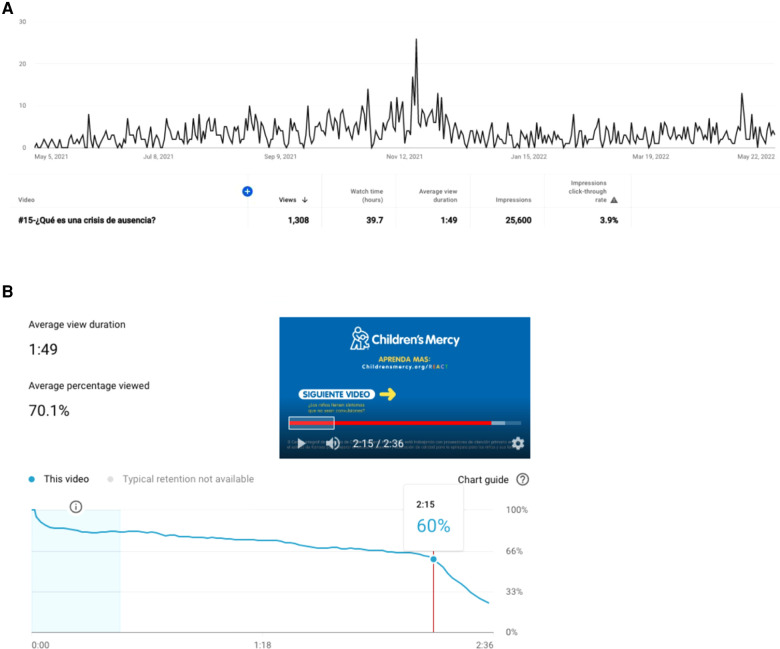
Example of a Spanish video analytics “¿Qué es una crisis de ausencia?” (What is an absence seizure?). (**A**) Views of top viewed video. The height of each peak represents the number of views during the indicated day. (**B**) Video audience retention. Average video viewing duration, red line marks beginning of the credits.

During the period evaluated, the REACT channel had 2,752 views, 91,300 impressions (presentation of video thumbnail) and a total watch time of 71.6 h. The highest source of traffic was YouTube search (40.5%), followed by suggested videos and external links, including those provided through the Children's Mercy web site (Table 2). The video with the highest traffic was “¿Qué es una crisis de ausencia?” [What is an absence seizure?], published May 5, 2021 with 1,308 views averaging 1:49 min (Figure 1A). This video had 25,600 impressions and a click-through rate of 3.9%. Traffic to this video originated primarily from YouTube search (54.2%) followed by “Suggested videos” (29.3%). The most common search terms leading to the video were “crisis de ausencia” (absence seizure—26.8%), “crisis de ausencia en niños” (absence seizure in children—6.3%) and “crisis de ausencia en adultos” (absence seizure in adults—4.1%). Location information for viewers is limited, but 12 views originated from Mexico and 11 from Peru. Traffic to the video has been consistent (Figure 1), with a spike of 26 views on Nov 18, 2021. The majority of traffic originated from mobile phones (74.3%), followed by computers (13.4%). The Spanish absence seizure video is 2:36 in duration. Average duration of view was 1:49. Viewers tended to remain engaged with the video until the credits at 2:15 into the content (Figure 1B).

**Table 2:** Views and traffic sources for the REACT YouTube channel.

Traffic source	Views (% of total)	Watch time (h)	Average view duration	Impressions	Impressions click-through rate (%)
Total	3,003	67.3623	0:01:28	91,300	1.99
YouTube search	1,159 (39)	31.2549	0:01:41	65,681	1.38
Suggested videos	526 (17.5)	14.0978	0:01:37	16,479	2.68
External	481 (16)	4.9905	0:00:57		
Browse features	260 (8.7)	6.7979	0:01:34	5,218	2.82
Playlists	201 (6.7)	2.887	0:00:51	2,093	8.22
Channel pages	134 (4.5)	3.0065	0:01:21	1,373	7.94
Direct or unknown	132 (4.4)	1.0288	0:00:37		
Other YouTube features	65 (2.2)	1.7986	0:01:42		
Playlist page	45 (1.5)	1.5003	0:02:00	456	8.11

Video analytics from the YouTube channel “REACT” where the 34 videos are located publicly.

To assess how these videos impact patients and their families we asked patients who were seen in one of our neurology clinics to complete the REDCap survey, as mentioned above. Of the 17 videos, 16 of the videos had at least one participant evaluation. A total of *n* = 40 people completed the demographics form and of these, *n* = 24 completed at least one video with the pre and post survey (Table 3). For the purpose of this data sample, we will focus on these 24 unique participants. This sample consisted of 75% of families having a CYE, 79% participating were Female, 71% reported Non-Hispanic/Latino ancestry, 79% reported White, 25% live in an urban setting, 42% in suburban setting, and 33% in a rural setting, and 87% reported having graduated from high school, 50% are married, 41.7% single and 8.3% divorced. Participants viewed an average of 2.6 videos (range 1–9 videos, Table 4). When we examined pre and post-test scores for all of the completed videos there was an average 28% improvement in correct response rate (range −33% to 100%).

**Table 3:** Educational video patient and family demographics.

Demographic characteristic	Full sample (*n* = 40)		No completed videos (*n* = 16)		Completed videos (*n* = 24)	
	Freq	%	Freq	%	Freq	%
Language preference						
English	40	100.0	16	100.0	24	100.0
Child with epilepsy						
No	8	20.0	2	12.5	6	25.0
Yes	32	80.0	14	87.5	18	75.0
Cargiver of child with epilepsy						
No	5	12.5	1	6.3	4	16.7
Yes	35	87.5	15	93.8	20	83.3
Gender identity						
Male	6	15.0	1	6.3	5	20.8
Female	34	85.0	15	93.8	19	79.2
Ethnicity						
Hispanic/latino	3	7.5	0	0.0	3	12.5
Non-hispanic/latino	33	82.5	16	100.0	17	70.8
Prefer not to answer	4	10.0	0	0.0	4	16.7
Race						
White	34	85.0	15	93.8	19	79.2
Black	3	7.5	1	6.3	2	8.3
Other	1	2.5	0	0.0	1	4.2
Prefer not to answer	2	5.0	0	0.0	2	8.3
Urban Status						
Urban	9	22.5	3	18.8	6	25.0
Suburban	22	55.0	12	75.0	10	41.7
Rural	9	22.5	1	6.3	8	33.3
Education						
Elementary school	1	2.5	0	0.0	1	4.2
Middle school	1	2.5	0	0.0	1	4.2
Some high school	1	2.5	0	0.0	1	4.2
High school or GED	14	35.0	5	31.3	9	37.5
Associate/technical degree	9	22.5	4	25.0	5	20.8
Bachelor's degree	10	25.0	7	43.8	3	12.5
Master's degree	4	10.0	0	0.0	4	16.7
Civil status						
Married	20	50.0	8	50.0	12	50.0
Single	16	40.0	6	37.5	10	41.7
Divorced	4	10.0	2	12.5	2	8.3

Demographic information for participants who completed the video and pre and post surveys.

**Table 4:** Patient/family educational video improvement scores.

Variable	*N*	Mean	Std Dev	Minimum	Maximum
Average improvement (% improvement)	24	0.28	0.28	−0.33	1.00
Average videos completed	24	2.63	2.24	1.00	9.00

Average improvement from the pre to post survey per video and average number of videos completed by each family/patient.

## Reaching the provider

While the ETG is significant in developed countries, it is exacerbated in LMIC. It is estimated that 80% of patients with epilepsy live in LMIC (5), yet these regions have the lowest access to proper diagnostic and management resources and physicians with adequate knowledge of epilepsy. This disparity in care is further intensified in rural regions (11, 12). The situation in Egypt with regards to epilepsy is representative of many LMIC. Egypt is classified by the World Bank as LMIC (https://data.worldbank.org/?locations=EG-XN). Egypt has an incidence of epilepsy of approximately 350,000 cases resulting in excess of 354,000 disability-adjusted life-years (a measure of disease burden calculated by adding years of life lost due to death and disability) (1). A cross-sectional study of children in the Al Kharga district and Al Queseir City found a prevalence of epilepsy of 9.7/1,000 with 59.4% of these cases being idiopathic, consistent with findings in other developing countries (13). Access to care remains limited, resulting in many patients suffering from regular breakthrough seizures. Egypt has a unique healthcare system in comparison to the United States. There are a total of four types of hospitals: university, Ministry of Health, military, and private. For this study we focused on university and military hospitals that are both private and public. University hospitals provide free health care services to anyone and free education to medical students, while military hospitals serve military personnel and their families (14). Providers who work for the government receive salaries from the public budget and those in the private sector are paid through performance-based incentives. There is no referral system and there is no existing structured electronic health record system (15).

### Method

Seven years ago, one member of the epilepsy team at Children's Mercy Kansas City (AA) developed several educational activities, both virtual and in-person, designed for neurology providers at several Egyptian universities, hospitals, professional organizations and the military all located in Cairo. This ongoing program consists of monthly virtual lectures presented on a variety of neurology and epilepsy topics such as: approach to the workup, comorbidities, pharmacological and non-pharmacological treatments, programmatic approach to organizing an epilepsy center, seizure precautions, handling familial and patient concerns, epilepsy surgery programs. These activities have multiple forms of engagement, including question and answer sessions, direct patient observations, workshops, conferences, symposia, virtual case studies, virtual and in-person lectures. Monthly virtual case reports presented by Egyptian providers offered an opportunity for constructive feedback. For data analysis purposes these activities were categorized as passive learning (e.g., virtual lectures) and active learning (e.g., case reports) (Table 5).

**Table 5:** Neurology provider activities.

Activities	Number of participants	Impact
	*N* (%)	Mean (STD)
Neurology virtual lectures	12 (80)	4.17 (0.58)
Neurology in-person lectures	11 (73)	4.45 (0.93)
Symposia	4 (27)	4.25 (0.50)
Conference session	12 (80)	4.33 (0.98)
PASSIVE LEARNING ACTIVITIES		4.22 (0.82)
Virtual case study sessions	10 (67)	4.60 (0.70)
Workshops (eg., EEG)	8 (53)	4.63 (0.52)
In-person patient visit observations	7 (47)	4.57 (0.53)
Direct contact for case-related inquires	10 (67)	4.90 (0.32)
ACTIVE LEARNING ACTIVITIES		4.65 (0.46)
**Changes based on impact of activities**	**Freq (%)**	
Somewhat	1 (7)	
A lot	5 (33)	
Extremely	9 (60)	

Learning activities completed by providers in Egypt. The first group of activity falls in the category of didactic or passive learning, while the second group required active participation. The right column is the participants ranking on a five-point Likert scale, with 5 being “extremely impactful” and 0 “not at all impactful”.

### Results

**Figure 2:**
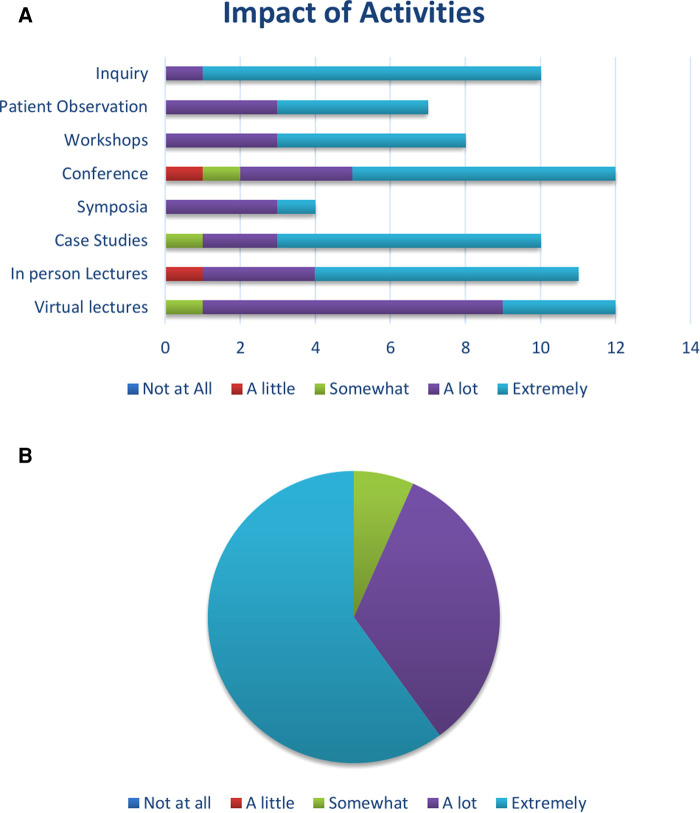
(**A**) Impact of activities on providers in Egypt. X axis is the number of physicians attending each activity. (**B**) Self-reported impact on clinical practice after participating in learning activities.

A cross-sectional survey of providers who attended the various activities was conducted from February to March 2022. Of the physicians, 40% are professors, 33% are assistant lecturers/specialists, 20% are lecturers/consultants and 7% are residents. Of the respondents, 53% identify as female and 47% as male. There is a broad representation of age, with 60% being between the ages of 26%–45% and 40% being between 36 and 65 years old. The physicians represent two universities and five hospitals including a military hospital. Years of experience in neurology varied with 40% having 4–9 years of experience and 60% having 10 or more years (*n* = 15, Table 6). The survey was created to measure provider knowledge and comfort levels on a variety of topics related to neurology/epilepsy as a result of participating in the educational activities. The survey consisted of qualitative and quantitative items. The quantitative data collected included: demographic information, number of neurology and epilepsy or seizure patients seen per month, and educational activity participation. The qualitative data included impact of educational activities on patient care, general knowledge and comfort level in managing different aspects of epilepsy, and interest in other topics for future educational opportunities. For the data analysis the teaching topics were categorized into four categories: treatment of epilepsy (11 sessions), epilepsy surgery (3), counseling (4) and routine management of epilepsy (4). The survey also included three open-ended questions. For each question, a conceptual analysis was conducted to quantify the presence of specific content categories through a process of selective reduction. The analysis was conducted based on these themes with flexibility to add categories as needed through the coding process (Table 7). Analysis revealed that participants in active learning activities (such as virtual case study sessions, workshops, in-person patient observations and direct contact for case-related inquiries) reported greater satisfaction than when engaging in passive learning activities (*M* = 4.65; SD = 0.46 compared to *M* = 4.22; SD = 0.82; Table 7, Figure 2A). Providers were then asked to rank their knowledge/comfort level in managing epilepsy patients on a 5-point Likert scale with 1 being “Extremely unknowledgeable/uncomfortable” to 5 being “Extremely knowledgeable/comfortable”. Participants were also asked to rank their colleagues’ overall knowledge regarding managing epilepsy patients. Interestingly, the participants generally ranked themselves higher than their colleagues (80% of physicians ranked themselves a 4 or 5 rating and only 20% ranked their colleagues a 4 or 5, Table 7). Overall, most providers reported that these activities resulted in significant changes in their practice, with over 90% of providers ranking the changes as “a lot” or “extremely” (Figure 2B, see also Table 7 for specific examples of practice changes following these training sessions).

**Table 6:** Egypt provider demographics.

	Freq	%
Rank		
Resident	1	6.67
Assistant lecturer/specialist	5	33.33
Lecturer (MD)/consultant	3	20
Professor	6	40
Sex		
Male	7	46.67
Female	8	53.33
Age		
26–35	6	40
36–45	3	20
46–55	4	26.67
56–65	2	13.33
Practice location		
University affiliation only	3	20
Hospital-based only	5	33.33
University and hospital	7	46.67
Years in neurology		
4–6 years	2	13.33
7–9 years	4	26.67
10 or more years	9	60
Specialty		
Pediatric neurology	3	20
Neurology	9	60
Neurophysiology	1	6.67
Neurosurgery	2	13.33
Percent epilepsy/seizure patients		
0%–10%	1	6.67
11%–20%	1	6.67
21%–30%	4	26.67
31%–40%	2	13.33
41%–50%	1	6.67
51%–60%	2	13.33
61%–70%	2	13.33
71%–80%	2	13.33

**Table 7:** Qualitative data quotes from providers in Egypt.

**What changes have you made in your practice from what you have learned? (Please specify. E.g., Is there a specific skill you gained?)**
*“I have better confidence at epilepsy practice”*
*“The whole way I dealt with EEG has change…it moved from being a diagnostic test into a complementary test for epilepsy patients”*
*“I became more skillful in EEG interpretation”*
*“Updated my knowledge of various epilepsy syndromes”*
*“Thorough understanding of the importance of data concordance in decision making in resective epilepsy surgery”*
**Why did you participate in the activities?**
*“Improve my skills, gain more experience and better practice”*
*“It is extremely helpful at practical and research levels”*
*“Because it was a rare opportunity to get such lectures from someone such as Dr. Ahmed”*
**What are you doing today in your practice that you didn't do prior? Please specify:**
*“Evaluation of drug resistant epilepsy patients”*
*“I look at epilepsy as a curable disease”*
*“Misdiagnosed typical absence as atypical”*
*“Improved EEG reading skills”*
*“Improved our battery for presurgical evaluation”*
*“Start to apply VNS and keto diet as treatment options for patients with DRE”*

Examples of direct quotes from the qualitative data section of the survey.

## Discussion

The ETG is a significant issue both in high- and LMIC. In the United States a retrospective study using a large database of almost 60,000 patients with a new epilepsy diagnosis revealed that up to one third of these patients remained untreated up to 3 years after epilepsy diagnosis (16). The situation with regards to access to care is exacerbated in rural areas. While the incidence of epilepsy in rural areas is similar to that of urban areas, access to care is not. In a study published by the Center for Disease Control in 2022, 7% of patients with active epilepsy reported not seeking care due to lack of transportation, and almost 6% had trouble finding a provider who would see them (17). These issues were exacerbated in lower income and underrepresented populations. Multiple other barriers prevent patients in rural areas from seeking care, including cultural perceptions and stigma, transportation, lack of access to services, and financial pressures (18). The situation in LMIC with respect to the ETG is much worse. In a report from the ILAE Epidemiology Commission the treatment gap was found to be as low as 5.6% in Norway and 10% in the USA, while it was greater than 50% in all of the LMIC examined (2). The COVID-19 pandemic resulted in a dramatic expansion of TH in the United States and around the world. This expansion in the practice of medicine seems to be here to stay and has resulted in a transformation of medical care (19). TH is a practical solution to reach patients with limited access to an epilepsy provider and is generally well accepted by both patients and providers (20). TH can be leveraged to address all aspects of care in epilepsy both in high- and LMIC (8). Kissani et al. set up a framework to reach both patients and providers using TH-based model called project ECHO (Extension for Community Healthcare Outcomes). The ECHO model has been adopted by the American Academy of Pediatrics for the treatment of epilepsy. Furthermore, the ECHO model has been applied successfully in 38 countries, in both high- and LMIC. In this paper we illustrate how this concept can be successfully applied, using three examples targeting different areas of need in the management of patients with epilepsy.

The first example is a case report of a patient with a mitochondrial disease and epilepsy living in the Kurdistan region of Iraq. By connecting experts in the United States and Iraq we were able to successfully start the patient on the ketogenic diet, resulting in a clinical improvement with reduced seizure burden, better weight gain, and improved cognition. This case shows that patients with complex diseases living in areas of the world with limited access to high level care can be successfully treated using telemedicine. This case is somewhat unique in that all the providers donated their time and expertise. Nevertheless, it serves to make the point that patients with drug resistant epilepsy can be successfully treated with the ketogenic diet.

In the second example of an application based on the ECHO model, we used virtual tools to provide education about epilepsy and seizures to families of CYE. Virtual tools can be synchronous (real-time), such as Zoom, or asynchronous (YouTube). Both are useful methods of TH that can be used for different necessities and audiences. Synchronous systems are very useful when a provider needs to communicate directly with patients, family members or fellow providers (as illustrated in the first example). When used to support remote locations, rural U.S. or international sites, these video systems may be limited by the technology infrastructure. Video resources describing clinical conditions can be a useful resource in support of TH. Educational videos can increase family engagement which will increase involvement in shared decision making with their providers, giving them more confidence in managing their child's condition. Using a series of 17 short videos available both in English and in Spanish, we demonstrate that using a dedicated YouTube channel provides multiple benefits. First, viewers beyond those directly associated with the organization providing care may benefit from the video resources. Of the REACT channel views during the period evaluated, 229 originated from our institution, Childrensmercy.org (Traffic source -> External), less than 10% of total views. YouTube is the second most utilized search engine (21). We found that 38.6% of project REACT views originated from a YouTube search. Notably, we found that the video with the most traffic was the Spanish version of the “What is an absence seizure?”. The majority of these views originated from YouTube search and users accessing the content from a mobile phone. The level of engagement was high, with most viewers watching the core content. The click-through rate for this video was nearly double that of the average for the entire channel, indicating a high level of interest in the content.

Designers of TH video content should consider using YouTube as a primary delivery platform as it expands the reach and impact of the content beyond the host organization. An investment in translating video content can result in meeting a need in under-served, under-represented communities in the U.S. and internationally. Examining a representative Spanish video demonstrated a high level of user engagement. Analysis of the search terms can guide the content designers to include key tags that may draw additional viewers to the content.

To further analyze the impact and applicability of including certain information in the educational videos, they were categorized into three groups and the average completion time per video was calculated for each group. Group 1 includes nine videos (1–7, 16 and 17) that provide general information regarding seizures and epilepsy. Of the *n* = 24 participants, 62 video surveys were completed and 50 of these came from the group 1. Therefore, each video in group 1 had an average of 5.6 completions. Group 2 includes six videos (8–13) that discuss the diagnosis or treatment plan options for epilepsy. Seven video completions came from group 2, meaning each video had an average of 1.2 completions. Group 3 includes two videos (14–15) that review *types* of seizures (tonic-clonic and absence). Six video completions came from group 3, meaning each video had an average of 3.0 completions.

There is a large variance in completion rates between the three groups of videos with group 1 having a higher completion rate. It is possible that these videos were viewed more frequently because the content is more generalized and applicable to CYE and their families. Group 2 covers videos that are more applicable to CYE with Drug Resistant Epilepsy (DRE), offering information on advanced treatment and non-pharmacological options such as: the Robotic Stereotactic Arm (ROSA), epilepsy surgery, ketogenic diet and neurostimulation. Approximately 7%–20% of CYE have DRE. It follows that a smaller portion of participants will view these videos as treatment options for their CYE (21). Finally, group 3 covers two specific types of seizures, absence and tonic-clonic seizures. Epidemiological studies have shown that 10% of CYE are diagnosed with absence seizures (22) and 50% of CYE and adults with epilepsy (23) are diagnosed with tonic-clonic seizures. Although the video on absence seizures had very few survey completions, this is the highest viewed video in Spanish according to our YouTube Analytics. This may be due to absence seizures being easily confused with other diseases such as ADHD, which may result in an apparent disproportionate interest in learning more about this topic.

For the third example of an application relying on virtual connections to address the ETG, we targeted epilepsy providers in a LMIC, Egypt. There are limited resources for neurology in most LMICs. For example, in Sub-Saharan Africa the median number of neurologists is 3 per 1 M population in comparison to 73 per 1 M in high-income countries. In addition, LMICs countries suffer from a lack of nurses and sub-specialized neurologic services, facilities for training, and struggle with access to treatments and antiepileptic drugs (22).

The survey shows providers ranked counseling patients and their families 3rd of 4 in comfort level, but paradoxically ranked it last of the topics they are interested in learning more about. Yet, proper counselling by the provider is a fundamental key in engaging patients and their families in effective treatment of CYE. It is an effective way to teach patients about their disease, introduce the risks associated with comorbidities, discuss sudden unexpected death in epilepsy (SUDEP), etc. We also found that providers are most comfortable with the routine management of epilepsy (ranked 1 in comfort level) but still, they expressed much interest in receiving more instruction on this topic, ranking second highest in the topics they want to hear more about. These findings reflect a greater interest in Egyptian providers to effectively treat epilepsy, and a much lower level of interest in addressing other psychosocial issues. There is clearly work to be done to emphasize the importance of addressing these issues for CYE. Not too surprisingly, we found that providers are the least comfortable with surgery related areas ranking it last, but they rank it first being the topic they are most interested in hearing more about. Surgery is the area with the largest gap but also the area that is the most “innovative” and “new” in a developing country. This opens real opportunities in a country ranked as a LMIC by the World Bank, to introduce new treatment modalities for epilepsy.

As part of the survey, providers were asked to rank both their own and their colleagues’ knowledge and comfort level in managing epilepsy patients. As noted in Table 8, 12 of the 15 providers ranked their own knowledge in the top two categories (somewhat or extremely knowledgeable/comfortable). This is in contrast to how they ranked their colleague's knowledge level, with only three providers ranking their colleagues’ knowledge as high, while 12 of the 15 providers ranked their colleague's knowledge as neural, somewhat or extremely unknowledgeable. There is no prior research regarding physician knowledge and comfort levels in managing epilepsy in Egypt. However, there was research conducted in neurosurgery training programs in Egypt. This study identified the three delivery models needed to improve effective neurosurgical treatment in Egypt: *Partnership or Twinning Model, On-Site Training Approach, and Online Neurosurgical Education*. It is reasonable to extend this model to neurology. The first approach, the *Partnership or Twinning Model*, relies on a longitudinal partnership between Egypt and high-income countries with a focus on a long-term collaboration between institutions in both countries, and regular site visits. The second practice model, the *On-Site Training Approach*, includes short-term visits, regular conferences, and workshops in the LMIC. The third practice model, the *Online Neurosurgical Education*, relies on virtual communication tools deepening the knowledge acquired in the first two practice models (14). We reprised these three practice models in the educational learning program we developed with Egypt, including developing long-term partnerships between institutions in both countries, brief onsite visits, and online neurological education.

**Table 8:** Providers self-ratings and ratings of colleagues.

Knowledge/comfort managing epilepsy patients	My own	My colleagues
Extremely unknowledgeable	0	1
Somewhat unknowledgeable	1	7
Neutral	2	4
Somewhat knowledgeable	11	2
Extremely knowledgeable	1	1

Physician comfort/knowledge scale in managing epilepsy for self and colleagues.

## Conclusion

While the ETG remains a problem both in high income countries, especially in rural areas, and in LMIC where it is anywhere from 5 to 10 times higher than in high income countries, the large increase in the use of TH is offering multiple opportunities to address this gap. In this paper we gave three examples of where TH was used to breach this gap. The first example showed that TH can be used effectively to treat patients with complex and rare disease in isolated regions. The second example illustrated the use of YouTube in asynchronous teaching to families of CYE located in rural areas. This model also illustrated that, given the wide availability of YouTube, these teaching videos were also effective at reaching patients well outside of our initial audience (this program was originally developed for CYE living in the state of Kansas), including many Spanish speakers. Finally, with the third example, we show that virtual tools can be effectively integrated within a comprehensive teaching program to reach providers in LMIC. There are many more similar programs with developing collaborations targeted to address the ETG both in underserved areas of the United States and in LMIC. With the marked use of TH that occurred following the COVID-19 pandemic, we are now in an ideal situation to aggressively address the ETG. It is our hope that this shift in how medical care is delivered will provide the foundation for more robust interventions worldwide, and subsequently result in a narrowing to the treatment gaps seen in most areas of medicine, including epilepsy.

## Data Availability

The original contributions presented in the study are included in the article/Supplementary Material, further inquiries can be directed to the corresponding author/s.
